# Retraction: Interactive effects of increased temperature, elevated pCO_2_ and different nitrogen sources on the coccolithophore *Gephyrocapsaoceanica*

**DOI:** 10.1371/journal.pone.0240917

**Published:** 2020-10-13

**Authors:** Citong Niu, Guicai Du, Ronggui Li, Chao Wang

After this article [1] was published, it came to light that there was a methodological error in preparing the filters for measurement of particulate organic carbon (POC), particulate organic nitrogen (PON), and particulate inorganic carbon (PIC). Recycled HCl was mistaken for concentrated HCl during removal of the coccolith layer, and as the recycled HCl had a relatively lower concentration the acid fume time of 12 hours was insufficient to remove the coccolith layer (inorganic carbon). Consequently, the reported PIC and POC measurements are not valid. This issue affects the results reported in Fig 2, Fig 3E, 3F, and Fig 4.

In light of this issue, statements in the article’s title, results and conclusions about individual and interactive effects of temperature, pCO_2_ and nitrogen source on the production rates of POC, PIC and PIC/POC are no longer supported. Therefore, the authors retract this article.

We would like to express our deepest apologies to the publisher and readers.
